# 2022 Editors’ Highlights

**DOI:** 10.1038/s42004-022-00795-0

**Published:** 2023-01-09

## Abstract

The Editors and Editorial Board of *Communications Chemistry* are pleased to launch an Editors’ Highlights Collection featuring some of our favorite Articles published in the journal in 2022. Here we highlight each Article and outline why it was selected.

Editorial Board Member Professor Jeff Chan highlights *A platinum(IV) prodrug strategy to overcome glutathione-based oxaliplatin resistance* by Christian Kowol, Walter Berger and colleagues (10.1038/s42004-022-00661-z). “High intracellular levels of glutathione characteristic of cancer cells are believed to be the main culprit of oxaliplatin drug resistance. In this study, the authors designed a unique platinum(IV)-based prodrug that features L-buthionine-S,R-sulfoximine appended as a ligand, which upon release can target and disrupt the biosynthetic pathway of glutathione.”

Editorial Board Member Dr François-Xavier Coudert highlights *Crystalline hydrogen bonding of water molecules confined in a metal-organic framework* by Nak Cheon Jeong, Dohyun Moon and colleagues (10.1038/s42004-022-00666-8). “Because a subtle balance of interactions governs the hydrogen bond network of water, its understanding in confined spaces of nanoscopic dimensions is a complex question, and its experimental characterization is full of challenges. The authors used a combination of in situ and ex situ synchrotron single-crystal X-ray diffraction and Raman spectroscopy to demonstrate that water in the nanocages of a paddlewheel metal–organic framework displays some crystalline characteristics, in both structure and dynamics, at room temperature.”

Editorial Board Member Professor Jun Lu highlights *Operando analysis of electronic band structure in an all-solid-state thin-film battery* by Ryoji Kanno and colleagues (10.1038/s42004-022-00664-w). “The team reveal the band structure of a model thin-film solid-state battery using operando hard X-ray photoelectron spectroscopy by treating this thin-film battery as a semiconductor device. This novel approach for the analysis of a model thin-film battery could deepen the understanding of battery reactions, thereby facilitating battery design and the development of battery protocols.”

Editorial Board Member Dr Satoshi Honda highlights *Electropolymerization without an electric power supply* by Shinsuke Inagi and colleagues (10.1038/s42004-022-00682-8). “Based on streaming potential generated in a microchannel, the team has succeeded in the electrochemical oxidative polymerization of aromatic monomers without the use of an electric power supply. This approach may open a new avenue toward an environmentally benign methodology for synthesizing a wide variety of molecules and polymers.”

Editorial Board Member Professor Wei Zhang highlights *Highly active and thermostable submonolayer La(NiCo)O*_*Δ*_ *catalyst stabilized by a perovskite LaCrO*_*3*_ *support* by Haiqian Wang and colleagues (10.1038/s42004-022-00686-4). “A trade-off between catalytic activity and structural stability generally exists in high temperature environments. The team synthesized a La(NiCo)O_Δ_ (LNCO) submonolayer catalyst (SML) stabilized by the surface lattice of a perovskite LaCrO_3_ support. Atomically dispersed Ni and Co atoms are anchored on the LNCO-SML surface lattice by strong Co–O bonds and defects. As a result, the LNCO-SML is highly active and very stable over 100 h in a stream test at 750 °C for the dry reforming of methane. Such results provide a vivid case, *via* optimal interface and surface modification, for designing atomically dispersed catalysts with high thermostablity.”

Editorial Board Member Professor Andy Wilson highlights *Controlling oncogenic KRAS signaling pathways with a Palladium-responsive peptide* by Eugenio Vázquez, José Mascareñas and colleagues (10.1038/s42004-022-00691-7). “This is a really nice interdisciplinary manuscript at the interface of chemical biology and supramolecular chemistry; it is beautifully presented and focuses as its centrepiece on the ability to reversibly nucleate an α-helix through coordination of histidine residues placed at the i and i + 4 positions in the peptide sequence with cis-protected palladium. The method is applied to target a key protein–protein interaction that activates the MAP kinase pathway—the interaction between RAS and its activator SOS1. RAS frequently misfunctions in human cancers and has become an important target for development of molecular therapeutics. What is particularly impressive about the paper is the broad range of experiments used; both circular dichroism and NMR are used for structure elucidation of the stimuli responsive peptide, then direct fluorescence anisotropy binding and nucleotide exchange assays used to characterize interaction with RAS and inhibition of the SOS1/RAS interaction, and then finally cell uptake is monitored alongside effects on the MAPK kinase cascade through inhibition of ERK phosphorylation. A further important concept is introduced, specifically that whilst the palladium clip induces some helicity in the peptide, this is only partial, but the effect is proposed to be sufficient to promote RAS binding (relative to the peptide in the absence of palladium) through a bind and fold mechanism similar to that through which many intrinsically disordered proteins operate. Overall, the paper comprises rigorous experimentation and introduces several new concepts. Given the widespread interest in the development of constrained peptides as protein–protein interaction inhibitors, this represents an important advance for the area of constrained peptides.”

Editorial Board Member Professor Yanli Zhao highlights *Bioinspired enzymatic compartments constructed by spatiotemporally confined in situ self-assembly of catalytic peptide* by Chunqiu Zhang, Deling Kong and colleagues (10.1038/s42004-022-00700-9). “The research team develops enzymatic compartments through a spatiotemporally controllable self-assembly of an artificial peptide in hollow porous glucan particles. Because of the formation of catalytic peptide nanofibers in the confined environment, the obtained enzymatic compartments exhibit enhanced substrate binding affinity as compared to the nanofibers in a dispersed system, along with a good stability against some perturbation conditions. This work demonstrates that enzymatic compartments can increase both the catalytic efficiency and lifetime of encapsulated enzymes.”

Chief Editor Dr Victoria Richards highlights *Global analysis of the energy landscapes of molecular crystal structures by applying the threshold algorithm* by Shiyue Yang and Graeme Day (10.1038/s42004-022-00705-4). “Current crystal structure prediction methods for anticipating polymorphism are lacking in their ability to provide information on the depth of each energy minimum and the possible transition paths and energy barriers between polymorph structures. Here, the team apply a Monte Carlo threshold algorithm to four known polymorphic organic systems in order to estimate the energy barriers that separate the different polymorph structures and characterize the global structure of the energy landscapes of their molecular crystal structures. Researchers who peer reviewed this work were excited that the method could potentially provide a solution to the over-prediction problem of crystal structure prediction, and that it could be used to check if metastable polymorphs can be kinetically stable. The peer review reports are available here.

Editorial Board Member Dr Jennifer Bridwell-Rabb highlights *Microbial rhodoquinone biosynthesis proceeds via an atypical RquA-catalyzed amino transfer from S-adenosyl-L-methionine to ubiquinone* by David Langelaan, Jennifer Shepherd and colleagues (10.1038/s42004-022-00711-6). “In this work, the team highlights an unusual role for *S*-adenosyl-L-methionine (SAM) in the enzyme RquA. SAM, which is traditionally recognized in biology for its ability to perform methylation or [4Fe-4S] cluster dependent radical chemistry, is instead shown to catalyze a Mn^2+^-dependent amino transfer reaction in rhodoquinone biosynthesis. This work thus reveals interesting SAM-dependent chemistry and contributes to our fundamental understanding of microbial rhodoquinone biosynthesis.”

Editorial Board Member Dr Kristin Wustholz highlights *Sample illumination device facilitates in situ light-coupled NMR spectroscopy without fibre optics* by Jack Bramham and Alexander Golovanov (10.1038/s42004-022-00704-5). “The team reports a new strategy to deliver light to solution-state NMR samples, without the need for fibre optics or probehead modifications, and demonstrate its capability for a variety of in situ photo-NMR studies. This “NMRtorch tube,” where the wall of the NMR tube acts as a light guide, could expand the accessibility of in situ photo-NMR experiments to include a wide range of existing NMR spectrometers.”

Editorial Board Member Dr Kui Yu highlights *Large emergent optoelectronic enhancement in molecularly cross-linked gold nanoparticle nanosheets* by Al-Amin Dhirani, Aftab Ahmed and colleagues (10.1038/s42004-022-00723-2). “The team reports an extended Langmuir approach to 2-dimension gold nanoparticle arrays that are molecularly cross-linked. The cross-linkers used include oligophenylene dithiol (HS-(C_6_H_4_)_n_-SH, *n* = 1, 2, and 3) that are conjugated and alkanedithiols (HS-(CH_2_)_n_-SH, *n* = 2, 4, 6, and 8). For the resulting hybrid organic molecule–metal nanoparticle sheets, their properties such as conductance and photoconductivity are studied. The reported cross-linking strategy is interesting with potential in the field of optoelectronics.”

Senior Editor Teresa Ortner highlights *Synthesis of rare-earth metal compounds through enhanced reactivity of alkali halides at high pressures* by Yuqing Yin and colleagues (10.1038/s42004-022-00736-x). High-pressure chemical synthesis—particularly explorative chemical synthesis—relies on the unreactivity of certain compounds such as crucible material. The chemical stability of alkali halides has caused them to be exploited as inert media in high-pressure, high-temperature experiments. Now, NaCl and KCl are unexpectedly found to react with yttrium, dysprosium and iron oxide in a laser-heated diamond anvil cell, producing Y_2_Cl, DyCl, Y_2_ClC, and Dy_2_ClC at ~40 GPa and 2000 K and FeCl_2_ at ~160 GPa and 2100 K.

Editorial Board Member Professor María Escudero-Escribano highlights *Tailoring the active site for the oxygen evolution reaction on a Pt electrode* by Masashi Nakamura and colleagues (10.1038/s42004-022-00748-7). “The team beautifully combines electrochemical methods with structural characterization using synchrotron-based X-ray diffraction on platinum single-crystalline electrodes to investigate the structure–reactivity relations during the oxygen evolution reaction. This work highlights the importance of carrying out model studies on well-defined surfaces to elucidate the active sites at the atomic level. The insight gained could be valuable for the rational design of more active and stable electrocatalysts for water electrolysis.”

Editorial Board Member Dr Carolina Horta Andrade highlights *Generative, and reinforcement learning approaches for the automated de novo design of bioactive compounds* by Maria Korshunova, Olexandr Isayev, and colleagues (https://www.nature.com/articles/s42004-022-00733-0). “The team use several technical innovations to address the issue of sparse rewards in reinforcement learning as the majority of the generated molecules are expectedly predicted as inactive. This is a fundamental computational and experimental study in the field of computer-assisted drug design and with an exemplary application of *de novo* design of new EGFR inhibitors, showing that there is no recipe for optimizing molecules through generative models, but when exploring the functions and hyperparameters, one can find the right protocol and that can be a powerful tool for designing new molecules with desired properties”.

Editorial Board Member Professor Hind Al-Abadleh highlights *Adsorption of oleic acid on magnetite facets* by Andreas Stierle, Robert Meißner and colleagues (10.1038/s42004-022-00741-0). “The team used a microscopic, experimental, and theoretical approach to investigate the magnetite/organic acid interface to illuminate processes responsible for tuning the mechanical properties of nanocomposite materials. They studied the adsorption and orientation of oleic acid molecules on two facets of magnetite crystals as a function of coverage under ultrahigh vacuum conditions and the results clearly showed the influence of surface termination on the structure of oleic acid surface complexes. The insights gained from the study are significant because they can guide future synthesis, modification, and stability of metal oxide nanocomposite materials in the presence of organic acids for optimum mechanical performance.”

Associate Editor Huijuan Guo highlights *Proton transfer during DNA strand separation as a source of mutagenic guanine-cytosine tautomers* by Louie Slocombe, Max Winokan, Jim Al-Khalili and Marco Sacchi (10.1038/s42004-022-00760-x). “The mechanism of point mutation during DNA replication is a long-standing biological question, and there are numerous discussions on the base-pair tautomerization during DNA duplex separation. This manuscript proposes the quantum chemical models of the separation of guanine-cytosine base pair using density functional theory and molecular dynamics simulations and finds increased stability of the tautomer when the DNA strands unzip as they enter a helicase enzyme, effectively trapping the tautomer population. This research paves the way to understanding the role of guanine–cytosine tautomerization on point mutation during DNA replication.”

Editorial Board Member Dr David Nelson highlights *Catalytic asymmetric synthesis of carbocyclic C-nucleosides* by Stephen Fletcher and colleagues (10.1038/s42004-022-00773-6). “The team have applied stereoselective rhodium catalysis to prepare a range of molecules that might then have future applications in chemical biology and medicinal chemistry. The work uses bench-stable arylboronic acids in the key step, and the resulting compounds are readily converted into C-nucleoside compounds with straightforward chemistry. The natural product Showdowmycin is prepared using this approach, and the authors note that many analogues might be prepared using this strategy.”

In addition to the many notable primary research Articles published in the journal this year, we are pleased to have published a number of timely and informative Review and Perspective articles, as well as valuable opinion pieces from the community in the form of Comment articles. It is our pleasure to highlight these contributions in our Collection alongside the above described research articles.

